# Epidural steroids following percutaneous endoscopic interlaminar discectomy: A protocol for systematic review and meta-analysis

**DOI:** 10.1097/MD.0000000000023531

**Published:** 2020-12-04

**Authors:** Qiang Ran, Yang Yu, Tong Li, Xiaohong Fan

**Affiliations:** a Chengdu University of Traditional Chinese Medicine; b Department of Orthopedics, Hospital of Chengdu University of Traditional Chinese Medicine, Chengdu, Sichuan Province, China.

**Keywords:** epidural steroids, percutaneous endoscopic interlaminar discectomy, protocol, systematic review

## Abstract

**Background:**

Percutaneous endoscopic interlaminar discectomy (PEID) has been widely used in the treatment of lumbar disc herniation and spinal stenosis, epidural steroids following PEID in an effort to reduce the incidence and duration of postoperative pain. Although steroids are widely thought to reduce the effect of surgical trauma, the observation index is not uniform, so the issue is still controversial. Therefore, the purpose of this paper is to systematically evaluate the efficacy and safety of local epidural steroids application following PEID.

**Methods:**

We will search the following databases from their inception to August 2020, PubMed, Embase, Medline, Chinese National Knowledge Infrastructure, Chinese Biomedical Literature Database, Web of Science, Wan Fang Database, Cochrane Library. The search strategy includes the MeSH terms. Meta-analysis will be performed using Rev Man V.5.3.5 statistical software.

**Results:**

This study will provide a high-quality synthesis to evaluate the efficacy and safety of local epidural steroids following PEID.

**Conclusion:**

This systematic review will provide evidence to judge whether local epidural steroids application following PEID is an effective and safe intervention for patients. It will provide reliable evidence for its extensive application.

**Registration number:**

INPLASY2020100085

## 1 Introduction

In patients with lumbar disc herniation and spinal stenosis, low back pain and sciatica are usually caused by the compression of the herniated disc by the nucleus pulposus.^[1]^ The conventional surgical technique for disc herniations is open discectomy with or without fusion surgery. By using minimally invasive treatment of lumbar disc herniation, percutaneous endoscopic Interlaminar discectomy (PEID) has been recognized by more and more clinicians.^[2]^ As a new form of therapy, PEID is to minimize the damage to the soft tissue of paravertebral muscles and the destruction of the bony structure of the lumbar vertebra. With rapid recovery following minimally invasive decompression surgery has been achieved, percutaneous endoscopic treatment of lumbar disc herniation may become the gold standard in the future.^[3]^

Up to 40% of patients after lumbar spine surgery experience recurrent persistent postoperative pain that may develop into long-term hospitalization.^[4]^ The physical compression of the nerve root by the herniated lumbar disc nucleus pulposus can be eliminated by surgery, but the postoperative inflammatory response and other stimulation may continue, persistent inflammation of the nerve may be the cause of persistent postoperative pain.^[5]^ Epidural injections for managing chronic low back pain are one of the most commonly performed interventions in the United States.^[6]^ There is a long history of local epidural steroids application in managing chronic low back pain and lower extremity pain of disc herniation or radiculitis.^[7]^ Epidural injections are administered by accessing the lumbar epidural space by multiple routes, including interlaminar, caudal, and transforaminal.^[8]^ But the local application of steroids in PEID remains controversial. However, few studies have investigated intraoperative local injection of steroids at the surgical site in an effort to reduce the incidence and duration of postoperative pain after PEID. This study aims to characterize the effect and safety of local intraoperative local epidural steroids application on perioperative and postoperative outcomes following PEID.

## 2 Method

### 2.1 Study registration

This protocol of systematic review and meta-analysis has been drafted under the guidance of the preferred reporting items for systematic reviews and meta-analyses protocols (PRISMA-P). Moreover, it has been registered on INPLASY (registration number: INPLASY2020100085).

### 2.2 Selection criteria

#### 2.2.1 Types of trials

Randomized controlled trials (RCTs) that evaluated the efficacy and safety of epidural steroids for the treatment PEID.

#### 2.2.2 Types of participants

Patients in chosen trials had been epidural steroids injected following PEID, providing appropriate management with outcome evaluations of 3 months or longer and statistical evaluations will be reviewed. Reports without appropriate diagnosis, nonsystematic reviews, book chapters, and case reports will be excluded.

#### 2.2.3 Outcome measures

The primary outcome parameter is relief of pain. The secondary outcome measure is functional status improvement. Postoperative complications will be observed in the meantime.

### 2.3 Exclusion criteria

#### 2.3.1 The exclusion criteria contain the following items:

(1) Non-RCTs reviews, case reports, expert experience, and conference articles.
(2) Incomplete data or information.
(3) Repeatedly checked or published literature.

### 2.4 Literature search

We will search the following databases from their inception to August 2020, PubMed, Embase, Medline, Chinese National Knowledge Infrastructure, Chinese Biomedical Literature Database, Web of Science, Wan Fang Database, Cochrane Library. The search strategy including the MeSH terms. PubMed strategies include a keyword search of non-Medline citations to retrieve in-process and supplied by publisher citations. The RCTs in English or Chinese associated with epidural steroids embedding for PEID will be included. There was no language restriction in the search. PubMed retrieval strategies are shown in Table 1.

**Table 1:** PubMed search strategy draft.

Number search item
#1 epidural space[MeSH Terms]
#2 steroids[MeSH Terms]
#3 #1 AND #2
#4 (“epidural”[All Fields] AND “space”[All Fields]) OR “epidural space”[All Fields] OR “epidural”[All Fields] OR “epidurally”[All Fields] OR “epidurals”[All Fields] OR “epiduritis”[All Fields]) AND (“steroidal”[All Fields] OR “steroidals”[All Fields] OR “steroidic”[All Fields] OR “steroids”[All Fields] OR “steroid”[All Fields])
#5 “Lumbar Vertebrae”[Mesh]
#6 Vertebrae, Lumbar[Title/Abstract]
#7 “diskectomy”[MeSH Terms]
#8 “Interlaminar”[All Fields] AND (“diskectomy”[MeSH Terms] OR “diskectomy”[All Fields] OR “discectomies”[All Fields] OR “discectomy”[All Fields])
#9 #4 OR#5 OR#6 OR#7 OR#8
#10 #3 OR #9
#11 (“percutaneous”[All Fields] OR “percutaneously”[All Fields] OR “percutanous”[All Fields]) AND (“endoscope s”[All Fields] OR “endoscoped”[All Fields] OR “endoscopes”[MeSH Terms] OR “endoscopes”[All Fields] OR “endoscope”[All Fields] OR “endoscopical”[All Fields] OR “endoscopically”[All Fields] OR “endoscopy”[MeSH Terms] OR “endoscopy”[All Fields] OR “endoscopic”[All Fields]) AND “Interlaminar”[All Fields] AND (“diskectomy”[MeSH Terms] OR “diskectomy”[All Fields] OR “discectomies”[All Fields] OR “discectomy”[All Fields])
#12 randomized controlled trial[Publication Type] OR randomized[Title/Abstract] OR placebo[Title/Abstract]
#13 #10 AND#11 AND#12

### 2.5 Data collection and analysis

#### 2.5.1 Data extraction and management

**Figure 1:**
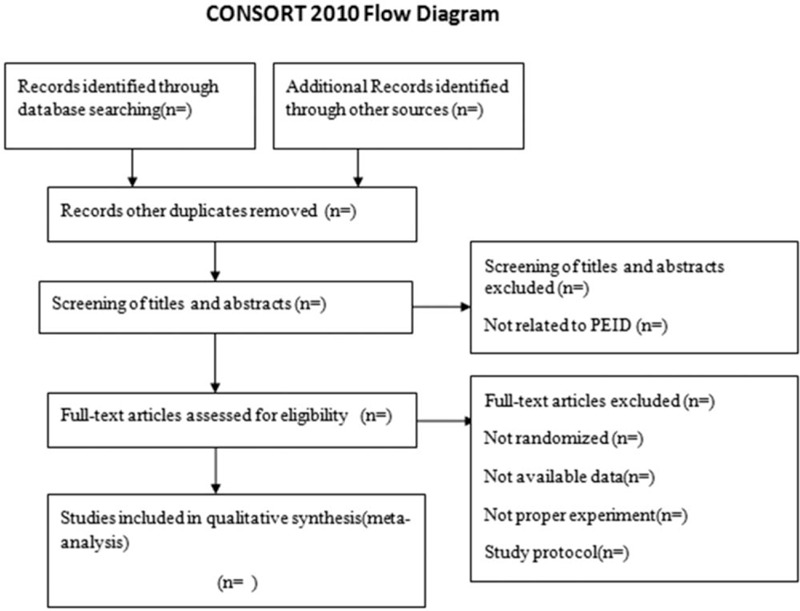
Flowchart of literature selection.

Two review authors (RQ and LT) independently searched for relevant literature, by reading titles, abstracts, and full texts, selected the manuscripts, and extracted the data from the included studies. Disagreements will be resolved by discussion between the 2 reviewers, if consensus could not be reached. The third reviewer will evaluate whether the studies will be satisfied according to inclusion criteria. The Grading of Recommendations Assessment, Development, and Evaluation approach will be used to evaluate the quality of evidence for all results of this systematic review. The quality will be divided into 4 levels: high, moderate, low, or very low. The diagram of this study is shown in Figure 1.

#### 2.5.2 Methodologic quality assessment

The literature of RCTs was evaluated with Cochrane bias risk assessment form, which included:

(1) the generation of random sequences;
(2) allocation concealment;
(3) blind method application;
(4) blind method evaluation;
(5) Data integrity;
(6) selective reporting of findings;
(7) other biases.

#### 2.5.3 Assessment of heterogeneity

In the analysis, isometric (*I*^2^) statistics are used to determine heterogeneity. The analysis of evidence is based on the presence of herniated discs or spinal stenosis to reduce any clinical heterogeneity.

#### 2.5.4 Assessment of risk of bias

The literatures will be reviewed with the Cochrane (Cochrane Manual V.5.1.0) Bias Risk Assessment. The funnel chart will be used to assess the reporting bias. When the number of studies is sufficient, we will use the Eger method to test.

The unit of analysis will be conducted by the independent reviewers. The missing data will be complemented by independent reviewers through contacting with the corresponding author. The funnel charts will be used to assess reporting biases.

#### 2.5.5 Sensitivity analysis

If necessary, the sensitivity analysis will be used to assess the effect of each study on the random effects model. Studies with a high risk of bias will be excluded to permit the evaluation of the robustness and reliability of the analysis.

#### 2.5.6 Subgroup analysis

We will observe the source of considerable heterogeneity by subgroup analysis based on variations in study and patient characteristics, study quality, different interventions, comparators, and outcomes.

#### 2.5.7 Statistical methods

Statistical analysis will be performed by Review Manager 5.3.3 (Cochrane Collaboration, Nordic Cochrane Centre, Copenhagen, Denmark). For the binary variables, the OR (odds ratio) and 95% CI (95% confidence interval) were used to evaluate the statistics, and for the continuous variables, the weighted mean difference (WMD) and its 95% CIs were used to analyze the statistics. For data extraction statistics, *I*^2^ is used to test the heterogeneity of the study. If *I*^2^ > 50%, the heterogeneity exists among the included studies. It is necessary to analyze the causes of heterogeneity from the data extracted from the literature. If *I*^2^ is less than 50%, homogeneity can be considered among the included studies.

## 3 Discussion

The pathogenesis of back and sciatica in patients with lumbar disc herniation are still unclear.^[9–12]^ The mechanical compression of the spinal nerve roots was thought to be the main cause of pain. Meanwhile, the nerve root inflammation plays a major role in the evolution of symptoms.^[13–15]^ With the development of instrumentation, PEID has been widely used to treat lumbar spine disease, but portion of patients still have short-term or long-term lumbar and sciatica postoperatively, which were related to nerve root inflammation. The PEID can completely expose the nerve root and the dura, the steroids can be used directly on the local place, which may reduce the patient's postoperative early stage of low back pain and leg pain by anti-inflammatory action.^[16,17]^ Shin et al^[18]^ reported epidural steroids application after PEID could reduce the back pain and sciatica, and functional outcomes were improved in the short-term postoperative period. In a prospective, randomized, single-blind trial, Chou et al^[19]^ reported local use steroids that did not lead to decreases in acute postoperative pain or narcotics consumption after discectomy. Aljabi et al^[20]^ reported local epidural steroids application could not decrease postoperative pain. So before steroids are routinely used by spinal surgeons, however, significantly more data are required from modern operating studies.

Intraoperative epidural steroids have been advocated for more than decades.^[21]^ The safety of epidural steroids has been demonstrated in both clinical and experimental; clinicians are concerned that epidural steroids could lead to infections. Overall, epidural steroids treatment seems to be quite safe.^[22]^ Sixteen trials were published from 1990 to 2012; none of the trials reported a significant increase of steroid-related complications.^[23]^ Although there were very few adverse effects reported in these RCTs, the safety of epidural steroids injections needs to be further evaluated.^[24]^

This article will be the first review on the systematic evaluation of intraoperative epidural application of steroid following PEID. It will draw reasonable conclusions by collecting evidence, sorting out and analyzing data. We hope this study will provide convincing evidence for both patients and clinicians.

## Author contributions

**Data collection:** Qiang Ran, Tong Li

**Formal analysis:** Xiaohong Fan, Qiang Ran

**Funding acquisition:** Xiaohong Fan

**Investigation:** Xiaohong Fan, Qiang Ran, Tong Li

**Software application:** Qiang Ran

**Supervision:** Xiaohong Fan

**Writing – original draft:** Qiang Ran

**Writing – review & editing:** Qiang Ran. Yang Yu
